# Study of the Relationship between Polymorphisms in the *IL-8* Gene Promoter Region and Coccidiosis Resistance Index in Jinghai Yellow Chickens

**DOI:** 10.3390/genes11050476

**Published:** 2020-04-27

**Authors:** Xiao-Hui Wang, Hai-Liang Yu, Wen-Bin Zou, Chang-Hao Mi, Guo-Jun Dai, Tao Zhang, Gen-Xi Zhang, Kai-Zhou Xie, Jin-Yu Wang

**Affiliations:** 1 College of Animal Science and Technology, Yangzhou University, Yangzhou, Jiangsu 225009, China; wxh9409161412@126.com (X.-H.W.); hailiangyu122514@163.com (H.-L.Y.); wenbinzou1216@163.com (W.-B.Z.); mch15861328120@163.com (C.-H.M.); zhangt@yzu.edu.cn (T.Z.); zgx1588@126.com (G.-X.Z.); yzxkz168@163.com (K.-Z.X.); jywang@yzu.edu.cn (J.-Y.W.); 2 Joint International Research Laboratory of Agriculture and Agri-Product Safety, the Ministry of Education of China, Yangzhou, Jiangsu 225009, China

**Keywords:** *Eimeria tenella*, interleukin-8 gene, promoter region, SNPs, resistance index

## Abstract

Interleukin 8 (IL-8) participates in the immune response and has the function of inducing neutrophils to release lysosomal enzymes and eliminate pathogens. This study was to investigate the effect of single nucleotide mutations in the *IL-8* gene promoter region on the coccidiosis resistance index. In this study, 180 infected *Eimeria tenella* (*E. tenella*) Jinghai yellow chickens were used as experimental samples. DNA sequencing technology was used to detect single nucleotide polymorphisms (SNPs) in the *IL-8* gene promoter region. The association between these SNPs and coccidiosis resistance indexes (including superoxide dismutase (SOD), malondialdehyde (MDA), glutathione peroxidase (GSH-PX), catalase (CAT), nitric oxide (NO), interleukin-1β (IL-1β), interleukin-2 (IL-2), interleukin-6 (IL-6), IL-8, and interferon-γ (IFN-γ)) were analyzed. Three SNPs (T-550C, G-398T, and T-360C) were detected. Significant associations were found between each genotype at the T-550C site with NO (*p*-value = 0.006) and IL-8 (*p*-value = 0.034) indexes. Significant associations were found between each genotype at the G-398T site with SOD (*p*-value = 0.042), CAT (*p*-value = 0.049), NO (*p*-value = 0.008), and IL-2 (*p*-value = 0.044) indexes. Significant associations were found between each genotype at the T-360C site with SOD (*p*-value = 0.007), NO (*p*-value = 0.046), IL-2 (*p*-value = 0.041), IL-8 (*p*-value = 0.039), and IFN-γ (*p*-value = 0.042) indexes. Haplotype analysis showed that multiple indexes of the H1H3 haplotype combination were significantly higher than other haplotype combinations. Therefore, mutation of the *IL-8* gene promoter region has a significant regulatory effect on the coccidiosis resistance index, with a change in transcription factor binding potentially altering *IL-8* gene expression, thereby further affecting the IL-8 level in plasma. However, the specific mechanism needs further study.

## 1. Introduction

Coccidiosis is a very serious intestinal disease caused by protozoan parasites, which can lead to significant weight loss, malnutrition, blood loss, increased susceptibility to other disease factors, reduced animal production performance, and massive economic losses to the poultry industry [1,2]. Chickens are susceptible to *Eimeria*, with *Eimeria tenella*, which is parasitic in the cecum, being one of the most pathogenic species. Pathological manifestations include intestinal mucosal injury, cecal swelling, bleeding, and discharge of bloody stools [3,4]. So far, control by drugs and live vaccines are the two major coccidiosis control strategies [5,6]. However, anticoccidial drugs and vaccination can cause risks, such as drug residues, drug resistance, and strain variation [7,8]. Therefore, studying the genes and single nucleotide polymorphisms (SNPs) associated with coccidia at the molecular level and applying the results to breeding new strains resistant to coccidiosis are effective ways to solve this problem. Interleukin 8 (IL-8) is a chemotactic cytokine that induces neutrophils to release lysosomal enzymes, clears pathogens, and also participates in immune responses, stimulating immune cell resistance [9,10,11]. Cornelissen et al. [12] showed that IL-8 can effectively recruit Th1 CD4+ cells, macrophages, and monocytes and then induce the production of interferon-γ (IFN-γ). Yu et al. [1] showed that glutathione peroxidase (GSH-PX), superoxide dismutase (SOD), catalase (CAT), and IL-2 play vital roles in the inflammatory response of chicken *E. tenella* infection. Swaggerty et al. [13] selected and bred broiler individuals with high levels of the leukocyte proinflammatory cytokines interleukin-6 (IL-6), IL-8, and chemokine chemokine c–c motif ligand 2 (CCLi2) for 3 consecutive generations and found that the ability of their offspring to resist *E. tenella* was significantly higher than that of a group with low expression levels. Lin [14] used RNA-seq technology to analyze the transcriptome of cecal tissues of *E. tenella*-infected chickens and non-infected control chickens and identified many significantly enriched pathways and differentially expressed genes, including *IL-6*, *IL-8*, interleukin-12β (*IL-12β*), interleukin-15 (*IL-15*), and transforming growth factor beta-2 (*TGFB2*). Min et al. [15] inoculated experimental chickens with pcDNA3-1E vaccine containing *IL-8* and *IL-15* genes, and found that the CD3+ lymphocytes increased significantly, and the number of fecal oocysts shed decreased significantly, which could be used as an adjuvant in the chicken emedia DNA vaccine. Ding et al. [16] injected recombinant protein (3-1e) in combination with cytokines such as IL-2, IL-8, and IFN-γ into chickens, and found that chickens could enhance their ability to resist coccidiosis infection. In this study, the aim was to examine the effect of mutations in the promoter region of the *IL-8* gene on coccidiosis resistance indicators and to use this information as a reference for breeding new coccidiosis-resistant chickens. 

## 2. Materials and Methods 

### 2.1. Experimental Animals

A total of 184 (92♂, 92♀) 1-day-old healthy Jinghai yellow chickens were selected from Jinghai Yellow Chicken Resource Farm (Haimen, China), housed in coccidiosis-free conditions and fed with antibiotic-free feed. All the chickens were free from parasitic infection, as tested by fecal detection, during the pre-test period. Each chicken was given an oral infection with 1.5 × 10^4^ *E. tenella* sporulated oocysts at the age of 30 days, and parasite oocysts were acquired from the Department of Parasitology, College of Veterinary Medicine, Yangzhou University. All animal protocols were approved by the Animal Welfare Committee of Yangzhou University (permit number: SYXK (Su) IACUC 2012-0029), and all efforts were made to minimize the suffering of the chickens. 

The method for counting the number of oocysts per gram of feces was as in [14]: 2 g of feces were mixed with 10 mL water and ground up. Saturated salt solution (50 mL) was added and mixed well. A small amount of fecal fluid was drained with a straw and added to the two counting rooms of the McMaster counter plate and counted under a microscope, the average was multiplied by 200 and repeated 3 times to give the number of oocysts per gram of feces. 

### 2.2. Sample Collection

Blood samples were collected from the wing vein of each bird on the 7th day post-infection, anticoagulated by heparin sodium, and centrifuged immediately at 4000 r/min for 5 min. After centrifugation, blood cells, and plasma were stored at −20 °C. 

### 2.3. DNA Extraction

The whole-blood genomic DNA of all chickens was extracted using the conventional phenol:chloroform extraction method. An Eppendorf BioPhotometer (Eppendorf Scientific, Hamburg, Germany) was employed to assess the DNA concentration and quality based on UV light absorbance at 260 and 280 nm. DNA samples of suitable quality were diluted to 100 ng/μL and stored at −20 °C.

### 2.4. Primer Design and PCR Amplification

**Figure 1:**
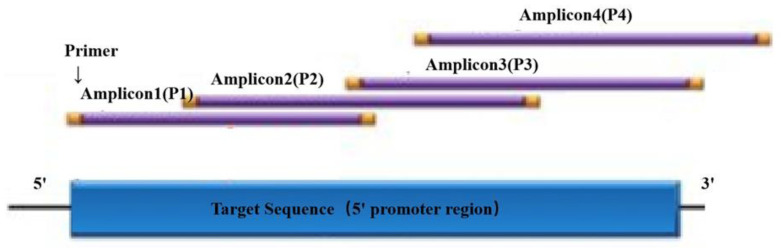
Amplicon layout in the promoter region of the interleukin 8 (*IL-8* ) gene.

According to the published chicken *IL-8* gene sequence (Gene ID: 396495) in GenBank, four pairs of primers were designed using Primer Premier 5 software for nucleotide sequence determination, ranging from −2000 bp to −1 bp upstream of the *IL-8* gene. The primers were synthesized by Sangon Biotech Co. (Shanghai, China). Primer sequences are shown in Table 1. The amplicon layout in the promoter region of the *IL-8* gene is shown in Figure 1. 

Polymerase chain reaction (PCR) was carried out as follows: 10 μL of 2 × Taq Master Mix, 1 μL each of the upstream and downstream primers, 1 μL of DNA template, and ddH2O to 20 μL were mixed. The PCR amplification procedures were: preliminary denaturation at 95 °C for 5 min; 35 cycles of denaturation at 95 °C for 30 s, annealing for 30 s at the optimum primer annealing temperature, and elongation for 1 min at 72 °C; and a final extension at 72 °C for 10 min. The samples were stored at 4 °C. 

### 2.5. SNP Identification

The purified PCR products were sequenced using a 3730XL DNA analyzer (Applied Biosystems, Foster City, CA, USA) at Sangon Biotech Co. We compared the sequencing results with DNAMAN 5.2 (Lynnon Corporation, San Ramon, CA, USA) and MEGA 6.06 to determine the SNPs of the *IL-8* gene in Jinghai yellow chickens. 

### 2.6. Transcription Factor Binding Site Prediction

AliBaba2.1 software [1] was employed to predict possible transcription factor binding sites (TFBS) in the promoter region of the chicken *IL-8* gene. During the prediction, the original sequence and the sequence containing the SNPs in the promoter region of *IL-8* gene were analyzed and changes to TFBS determined.

### 2.7. Linkage Disequilibrium and Haplotype Analysis

SHEsis analysis software [1] was used for linkage disequilibrium and haplotype analysis of the identified SNPs in the promoter region. 

### 2.8. Resistance Index Detection

Plasma antioxidant indexes were determined by biotin double antibody sandwich enzyme-linked immunosorbent assay (ELISA) according to the manufacturer’s instructions (Institute of Bioengineering, Nanjing) [14,17]. These indexes included superoxide dismutase (SOD), malondialdehyde (MDA), glutathione peroxidase (GSH-PX), catalase (CAT), nitric oxide (NO), interleukin-1β (IL-1β), interleukin-2 (IL-2), interleukin-6 (IL-6), IL-8, and interferon-γ (IFN-γ). 

### 2.9. Statistical Analysis

The associations of the SNPs and haplotype with the *E. tenella* resistance index in Jinghai yellow chickens were analyzed by the least square model:

Y=μ + Sex + Genotype + (Sex × Genotype) + e

where Y is the value of the resistance index for each bird; μ is the overall mean; Sex is the effect of sex; Genotype is the effect of genotype or haplotype combination; Sex × Genotype is the interaction effect between sex and genotypes; and e is the random error effect. 

The *F* test of each factor and interaction between factors were performed by using SPSS18.0 software (IBM, NK, USA) [18]. 

The LSD multiple comparison method was used to compare the difference among groups [18]. The method of multiple testing correction was the Bonferroni adjustment. Statistical significance occurred at *p*-values < 0.05. 

## 3. Results

### 3.1. Clinical Observation

On days 1–3 post-infection, the infected chickens had no noticeable symptoms. On the fourth day post-infection, the infected chickens began to excrete bloody diarrheas, and particularly on the fifth day after infection, bloody diarrheal excretion was the highest. On days 6–7 post-infection, bloody diarrheal excretion began to decrease. The infected chickens appeared listless, wasted food, and presented anal filth along with paralytic and symptoms of spasm. No chicken deaths occurred during the trial period.

### 3.2. SNP Identification in IL-8 Promoter

**Figure 2:**
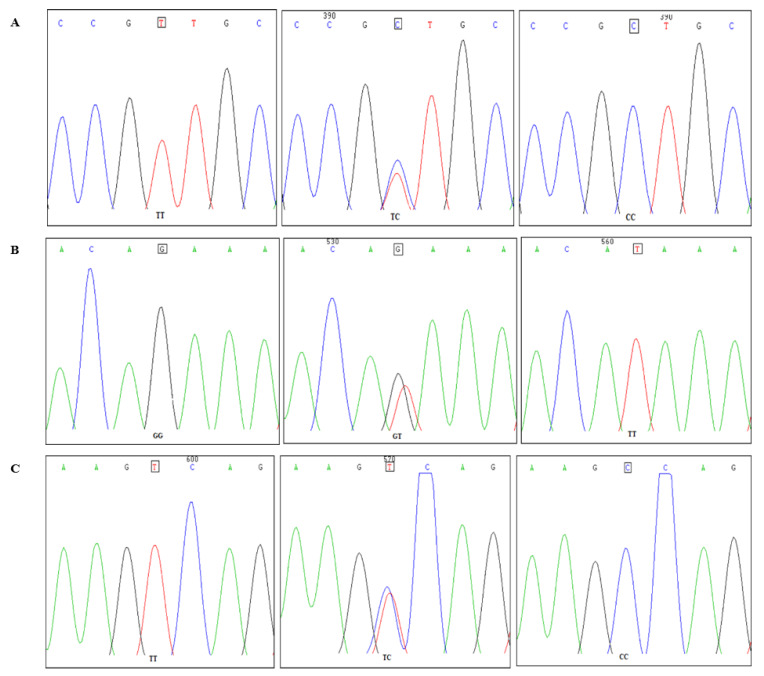
Analysis result chart of 3 SNPs site. (**A**): T−550c peak alignment; (**B**): G−398T peak alignment; (**C**): T−360C peak alignment.

Three SNP sites were detected by sequencing and sequence alignment. Mutation site 1 was a T mutated to C at –550 bp (T–550C), the chromosome location was 51282560 and the chromosome (rs) number was rs740065165. Site 2 was mutated from G to T at –398 bp (G–398T). It was located at 51282712 in the chromosome, and the rs number was rs731947764. Site 3 was mutated from T to C at –360 bp (T–360C), which was located at 51282750 in the chromosome, and the rs number was rs16409254. The sequencing results of the 3 SNPs are shown in Figure 2.

### 3.3. Prediction Results for Transcription Factor Changes Caused by Mutations in SNPs in the Promoter Region of the IL-8 Gene

As shown in Table 2, a mutation from T to C occurred at −550 bp in the promoter region of the *IL-8* gene, where the mutation caused the original Oct-1 transcription factor binding site to disappear. A mutation from G to T occurred at −398 bp, resulting in a positional change in the binding site of the original C/EBPalp transcription factor, and a binding site for a Pit-1a transcription factor was added. 

A mutation from T to C at −360 bp resulted in the disappearance of the original NF-1 transcription factor binding site. 

### 3.4. Association Analysis of SNPs in the IL-8 Gene Promoter Region and Plasma Resistance Indexes

#### 3.4.1. Association Analysis between Each Genotype of the T–550C Mutation and Coccidiosis 

##### Resistance Indexes of Chickens

**Figure 3:**
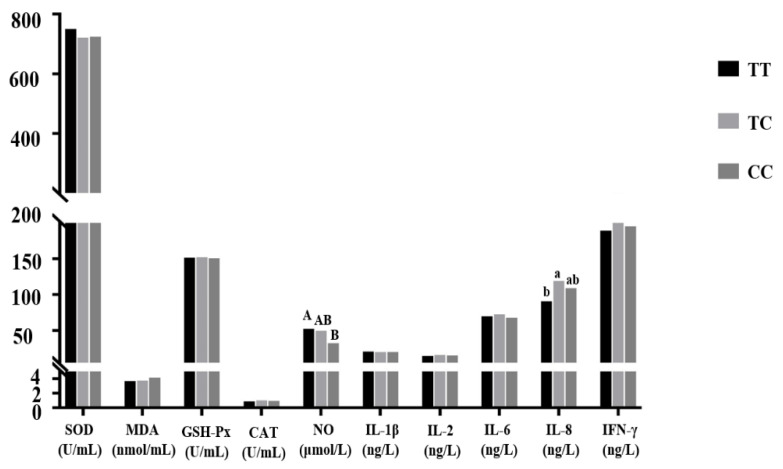
Correlation analysis between each genotype of the *IL-8* gene T–550C mutation and coccidiosis resistance indexes. Note: Different uppercase letters in the same indexes indicate significant differences (*p* < 0.01); different lowercase letters in the same indexes indicate significant differences (*p* < 0.05); the same or no letters indicate no significant differences (*p* > 0.05). The number of individuals of each genotype is shown in brackets. The same representation is used in Figure 4, Figure 5, Figure 6 and Figure 7.

In all the figures, different uppercase letters in the same indexes indicate significant differences (*p* < 0.01); different lowercase letters in the same indexes indicate significant differences (*p* < 0.05); the same or no letters indicate no significant differences (*p* > 0.05). The number of individuals of each genotype is shown in brackets. As shown in Figure 3, except for the SOD, MDA, NO, and IL-1β indexes, the other indexes of TC genotype individuals were higher than those of chickens with the other two genotypes, but the difference was not significant (*p* > 0.05). The IL-8 index of TC genotype individuals was significantly higher than that of TT genotypes (*p* < 0.05); the NO index of TT genotype individuals was significantly higher than that of CC genotype chickens (*p* < 0.01)

#### 3.4.2. Association Analysis between Each Genotype of the G–398T Mutation and Resistance Indexes of Chicken Coccidiosis

**Figure 4:**
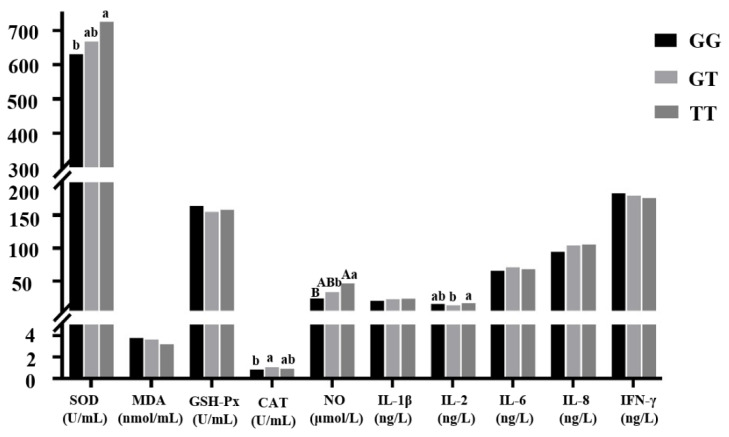
Correlation analysis between each genotype of the *IL-8* gene G–398T mutation and coccidiosis resistance indexes.

As shown in Figure 4, the SOD index of TT genotype individuals and the CAT index of GT genotype individuals were significantly higher than those of GG genotype chickens (*p* < 0.05). The NO index of TT genotype individuals was significantly higher than that of GG genotype chickens (*p* < 0.01) and significantly higher than that of GT genotype chickens (*p* < 0.05). The IL-2 index of TT genotype individuals was significantly higher than GT genotype chickens (*p* < 0.05). The IL-1β and IL-8 indexes of TT genotype individuals were higher than those of GG genotype chickens, but the differences were not significant (*p* > 0.05). 

#### 3.4.3. Association Analysis between Each Genotype of the T–360C Mutation and Coccidiosis 

##### Resistance Indexes of Chickens 

**Figure 5:**
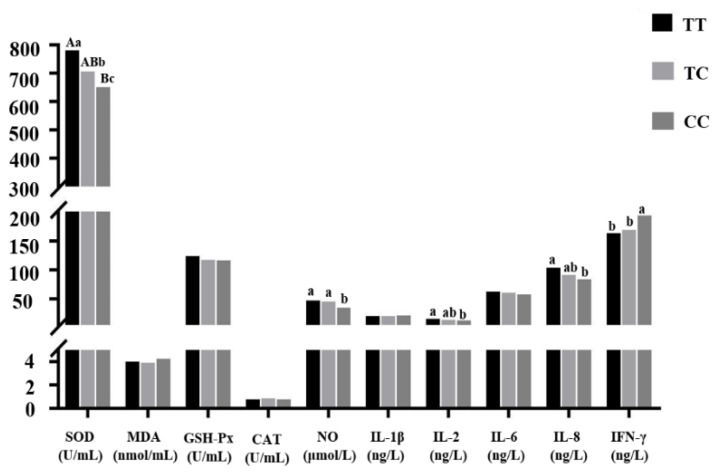
Correlation analysis between each genotype of the *IL-8* gene T–360C mutation and coccidiosis resistance indexes.

As shown in Figure 5, except for the MDA, CAT, IL-1β, and IFN-γ indexes, other indexes of TT genotype individuals were higher than those of chickens with the other two genotypes. For the SOD index, the difference between TT genotypes and CC genotypes was significant (*p* < 0.01), and TC and CC genotypes were significantly different (*p* < 0.05). Both TT and TC genotype individuals had significantly higher NO indexes than CC genotype chickens (*p* < 0.05). TT genotype individuals had significantly higher IL-2 and IL-8 indexes than CC genotype chickens (*p* < 0.05). CC genotype individuals had significantly higher IFN-γ indexes than the chickens with the other two genotypes (*p* < 0.05). The GSH-PX and IL-6 indexes of TT genotype individuals were higher than those of the chickens with the other two genotypes, but the differences were not significant (*p* > 0.05). 

### 3.5. Linkage Disequilibrium Analysis of Each Mutation Site in the IL-8 Gene Promoter Region

**Figure 6:**
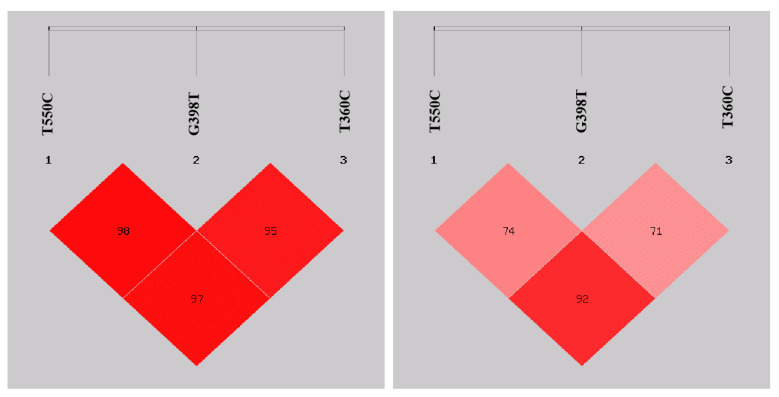
D’ values (**left**) and r^2^ values (**right**) of pairwise linkage disequilibrium analysis of the *IL-8* gene promoter region.

D’ and r^2^ are two commonly used parameters to measure linkage disequilibrium. The probability of the occurrence of recombination events in the linkage disequilibrium region can be directly reflected by the size of D’, while the effectiveness of correlation analysis is directly related to r^2^. Because D’ is insensitive to changes in gene frequency, r^2^ is more reliable than D’ when one gene at one site is less frequent [19]. The result is shown in Figure 6. The graphical representation, generated by SHEsis, of the r^2^ linkage disequilibrium relationship between each SNP is expressed in different colors, where deep red represents high linkage disequilibrium (r^2^ > 0.80) and light red represents low linkage disequilibrium (r^2^ < 0.80). The r^2^ value for each pair-wise comparison is shown (%), indicating a strong linkage disequilibrium between the T–550C and T–360C mutations.

### 3.6. Haplotype Analysis and Association Analysis between Haplotype Combinations and Resistance Indexes of the IL-8 Gene Promoter Region Mutation Sites

**Figure 7:**
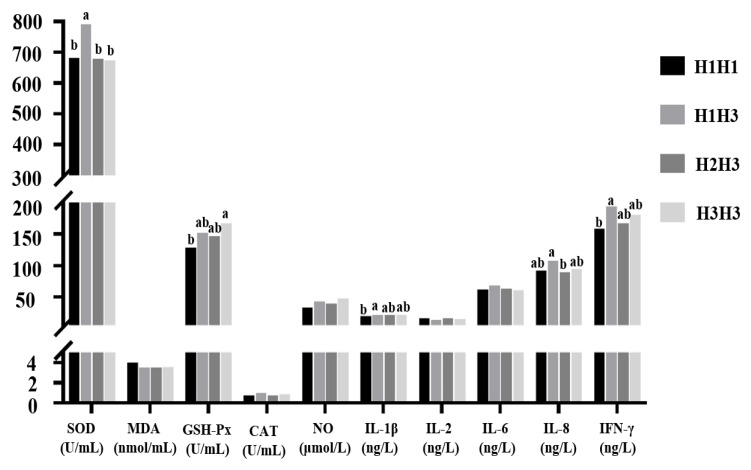
Effect of different haplotype combinations of the *IL-8* gene on resistance indexes.

The haplotype analysis produced three haplotypes, H1 (CGC), H2 (TGT), and H3 (TTT), with frequencies of 30.4%, 5.4%, and 61.4%, respectively. Six haplotype combinations (H1H1, H1H2, H1H3, H2H2, H2H3, and H3H3) were produced. The haplotype combination effect on coccidiosis resistance indexes is shown in Figure 7 (haplotype combinations with less than 3 individuals were not used in multiple comparisons). The results showed that the SOD content of H1H3 was significantly higher than that of the other three haplotype combinations (*p* < 0.05). The GSH-PX content of H3H3 was significantly higher than that of the H1H1 haplotype combination (*p* < 0.05), and there were no significant differences between H3H3 and H1H3 or H2H3 (*p* > 0.05). The IL-1β and IFN-γ contents of the H1H3 haplotype combination were significantly higher than those of the H1H1 haplotype combination (*p* < 0.05), and there were no significant differences between H1H3 and H2H3 or H3H3 (*p* > 0.05). The IL-8 content of the H1H3 haplotype combination was significantly higher than that of the H2H3 haplotype combination (*p* < 0.05), and there were no significant differences between H1H3 and H1H1 or H3H3 (*p* > 0.05). 

## 4. Discussion

### 4.1. Prediction of Transcription Factor Binding Sites in the IL-8 Gene Promoter Region

The promoter region is the core region of gene transcription regulation, and the SNPs formed by mutation in this region can change the original transcription factor binding sites and associated transcription factors, thereby affecting the expression of traits related to the gene [20]. A transcription factor binding site is a DNA sequence recognized and bound by a transcription factor, with the transcription process being regulated by the interaction between the two. If the binding site changes, it will have an important influence on the binding of transcription factors to regulatory sequences and the expression products of the genes. Therefore, the study of SNPs in gene promoter regions and their association with related traits is also an important way to discover new molecular genetic markers [21,22]. Korkaya et al. [23] found that activated nuclear factor-kappa B (NF-κB) has a positive regulatory effect on the expression of IL-6 and IL-8 in epithelial cells. Wu et al. [24] showed that inhibition of activating transcription factor 3 (ATF3) significantly increased the expression of the proinflammatory cytokines IL-6 and IL-8 in human bronchial epithelial cells. Octamer transcription factor (Oct-1) is a member of the Pit-Oct-Unc (POU) homeodomain family. Oct-1 binds to a diverse range of cis-regulatory DNA sequences through its DNA-binding POU domain and can carry on the negative regulation to the gene [25]. CCAAT enhancer binding protein (C/EBPalp) and nuclear factor-1 (NF-1) play important roles innumerous cellular responses by regulating the transcription of genes from target cells, including the cell proliferation and differentiation, tumorgenesis and apoptosis, and cell cycle regulation [25,26]. Pituitary specific transcription factor (Pit-1a) can bind to and transactivate promoters of growth hormone- and prolactin-encoding genes, and has an important regulatory effect on animal growth and immunity [27]. The transcription factor prediction results of the *IL-8* gene promoter region in this experiment indicated that the mutation of T to C at −550 bp caused the original Oct-1 transcription factor binding site to disappear. The mutation of G to T at −398 bp resulted in a positional change in the binding site of the original C/EBPalp transcription factor, and a binding site for the Pit-1a transcription factor was added. The mutation of T to C at −360 bp resulted in the disappearance of the original NF-1 transcription factor binding site. These three mutations have changed the original transcription factor interaction, thereby potentially regulating promoter activity. Changes in transcription factors may change the expression of the *IL-8* gene, which in turn affects the content of IL-8 in plasma. IL-8 is involved in the immune response and plays an important role in inflammation and the immune response. Studies have shown that the higher the expression of IL-8 in the body is when chickens are infected with coccidia, the stronger the resistance to coccidiosis [13]. Therefore, it is speculated that the change in transcription factors may affect the chicken’s ability to resist coccidioides, but the specific mechanism remains to be further studied. 

### 4.2. Relationship between SNPs in the IL-8 Gene Promoter Region and Resistance Indexes of Chicken Coccidiosis 

Under normal circumstances, the body’s antioxidant enzymes are in a dynamic equilibrium. When the body’s steady state is destroyed, these enzymes will change to maintain the balance of the body’s antioxidant system [28,29]. Cytokines are proteins secreted by immune cells that regulate immune responses through intercellular interactions [30]. Therefore, improving the resistance index of chickens can be an important way to resist coccidiosis infection. In this study, the *F* test showed that there were no significant effects between sex, and interactions between sex and genotype. SOD, CAT, GSH-PX, and MDA constitute the body’s antioxidant system. CAT is an oxidoreductase capable of decomposing hydrogen peroxide, GSH-PX can work with SOD to remove active oxidative free radicals, and MDA can degrade lipid peroxidationproducts [1,14]. For the T-550C mutation, GSH-PX, CAT, IL-2, IL-6, and IL-8 indexes of TC genotype individuals were significantly different from those of chickens with the other two genotypes. NO can inhibit or kill parasites. Il-2 and other cytokines in the chicken body can induce the secretion of IgA antibodies in immune cells and enhance the immune ability to diseases [14]. For the G-398T mutation, the SOD, CAT, NO, and IL-2 indexes of TT genotype individuals were significantly different from those of chickens with the other two genotypes. IFN-γ can improve the level of the chicken’s protective cellular immunity against coccidiola [14]. For the T-360C mutation, the SOD, NO, IL-2, IL-8, and IFN-γ indexes of TT genotype individuals were significantly different from those of chickens with the other two genotypes. After cocciditis infection, cell metabolism and dysfunction will be caused with the aggravation of organ and tissue damage, and the changes of these indexes may be a manifestation of the body playing a defense role to reduce the damage of the body. The results of Lin and Yu et al. demonstrate this [1,14]. In this study, compared with other genotypes, individuals with certain genotypes formed by mutation have higher anticoccidial ability, and the selection of such individuals may be beneficial to the improvement the population’s anticoccidial abilities. 

### 4.3. Linkage Disequilibrium and Haplotype Analysis

Linkage disequilibrium usually occurs in natural populations, and sites that are in linkage disequilibrium tend to be inherited as a whole in haplotypes [31,32]. Studies have shown that multiple-site combined haplotype analysis considers the interaction of linkage disequilibrium and non-allelic inheritance between SNPs, which has enhanced statistical power [33]. The results showed that the H1H3 haplotype combinations were higher than other haplotype combinations in the resistance indexes for CAT, IL-1β, IL-6, IL-8, IFN-γ, and the SOD content of the H1H3 haplotype combination was significantly higher than the other three haplotype combinations. Thus, the H1H3 haplotype combination is a dominant haplotype combination, which can provide a reference for the breeding of new coccidium-resistant strains. In this study, we detected 14 SNPs in the promoter region of the *IL-8* gene. Among the 14 SNPs detected, only three SNPs were significantly correlated with the selected resistance indexes. These associations need to be further tested for their correlation with larger populations. Because these SNPs have the potential to change gene expression, this study can be classed as a preliminary study paving the way for future in-depth studies. 

Three mutation sites were detected in the promoter region of the *IL-8* gene in Jinghai yellow chickens, which changed their original transcription factor interactions. Correlation analysis with coccidioides resistance indexes revealed that the genotypes of the SNPs and dominant haplotypes in the promoter region of the *IL-8* gene have significant effects on resistance indexes. This result indicated that mutation of the *IL-8* gene promoter region related to the coccidioides resistance index may affect *IL-8* gene expression, but the specific mechanism needs further study. 

## Figures and Tables

**Table 1:** Primer sequence information.

Primer	Primers Sequence (5′→3′)	Annealing Temperature	Length (bp)
P1	F: TTCCATTCGCATAAGTCATC R: AAAGTTGATTTGGGGATACC	51 °C	638
P2	F: TGTAATTGGGAATTCAAGGGGGA R: CCCATTTGGTGTGTGATAAGATGA	58 °C	708
P3	F: AGTCCACAGACCACAAAGCA R: TCGCAATATAAGTTTCTGATGGCTT	58 °C	693
P4	F: AAACCAGCAACACAAAGTC R: CATCTCAGCAAGTGCCAAG	60 °C	574

**Table 2:** Prediction results for transcription factor changes before and after single nucleotide polymorphisms (SNP) mutations in the 5’ regulation region of the *IL-8* gene.

Mutation Site	Base	Transcription Factor	Transcription Factor Binding Site Base Sequence	Transcription Factor Position
−550	T C	Oct-1	gttgcatttg	−551~−542
−398	G	C/EBPalp	gaaataaata	−398~−389
	T	Pit-1a	taaataaata	−398~−389
		C/EBPalp	acataaataa	−401~−392
−360	T C	NF-1	agccagttat	−362~−353

Note: The underlined letters in the table indicate mutated bases.
